# Tumor treating fields enhance anti-PD therapy by improving CCL2/8 and CXCL9/CXCL10 expression through inducing immunogenic cell death in NSCLC models

**DOI:** 10.1186/s12885-025-13859-w

**Published:** 2025-03-17

**Authors:** Wei Lin, Yingying Wang, Minghao Li, Jingjing Feng, Ying Yue, Jing Yu, Yanjiang Hu, Yuanzhen Suo

**Affiliations:** 1 Department of Rheumatology and Autoimmunology, The First Affiliated Hospital of Shandong First Medical University & Shandong Provincial Qianfoshan Hospital, Jinan, 250014 China; 2 School of Clinical and Basic Medicine, Shandong First Medical University & Shandong Academy of Medical Sciences, Jinan, 250117 China; 3 Shandong Key Laboratory of Rheumatic Disease and Translational Medicine, Shandong Medicine and Health Key Laboratory of Rheumatism, The First Affiliated Hospital of Shandong First Medical University, Jinan, 250014 China; 4 Department of Critical-care Medicine, Shandong Provincial Hospital Affiliated to Shandong First Medical University, Jinan, 250021 China; 5 Healthy Life Innovation Medical Technology Co., Ltd, Wuxi, 214174 China; 6 Department of Thoracic Surgery, Liyang People’s Hospital, Liyang, 213300 China; 7 Liangzhu Laboratory, Zhejiang University, Hangzhou, 310058 China

**Keywords:** Non-small cell lung cancer, Tumor treating fields, anti-PD immunotherapy, T cell infiltration, Immunogenic cell death, Chemokines

## Abstract

**Background:**

Non-small cell lung cancer (NSCLC) is the most common type of lung cancer. Tumor treating fields (TTFields) combined with anti-PD immunotherapy offers a promising strategy to address this issue. Nevertheless, the mechanism of action (MOA) of TTFields therapy combined with anti-PD immunotherapy in NSCLC has not been thoroughly investigated. This study aims to elucidate the MOA of the combined therapy from the aspect of improving the tumor immune microenvironment (TIME).

**Methods:**

Using a mouse model of NSCLC, we tested the efficacy of TTFields therapy with anti-PD-1 and anti-PD-L1 immunotherapy. By RNA-seq, the differential genes and signaling pathways between combination therapy and anti-PD therapy groups were studied. In-vitro experiments validated the effects of TTFields on tumor cells for CD4^+^ T cell and CD8^+^ T cell infiltration, as well as the expression of tumor immunogenic death related genes and chemokines.

**Results:**

Combining TTFields with anti-PD-1 reduced tumor weight and volume, respectively, compared to controls (*p* < 0.05). RNA-seq analysis revealed 1,745 differentially expressed genes (DEGs) in the combination therapy group versus controls, including upregulated immune pathways and immunogenic cell death (ICD) associated genes. Further study showed that the combination therapy resulted in increased T cell infiltration compared to anti-PD immunotherapy alone, and TTFields induced higher level expression of ATP, HMGB1, CCL2, CCL8, CXCL9, and CXCL10 and inflammatory cytokines than control group. These effects collectively contributed to the altered TIME, and finally potentiated the efficacy of anti-PD therapy.

**Conclusions:**

TTFields enhance the effectiveness of anti-PD immunotherapy by improving CD4^+^ T cells and CD8^+^ T infiltration via inducing ICD to increase CCL2/8 and CXCL9/CXCL10 expression of tumor cells. This study provides theoretical basis and new insights for evaluating the effectiveness of TTFields combined with anti-PD therapy for NSCLC.

**Supplementary Information:**

The online version contains supplementary material available at 10.1186/s12885-025-13859-w.

## Introduction

Lung cancer stands as a leading cause of cancer-related deaths worldwide, accounting for 18.7% of all cancer fatalities [1]. Non-small cell lung cancer (NSCLC) comprises 85% of all lung cancer cases [2]. The transformative success of targeted therapies and immunotherapies has profoundly altered the treatment landscape of NSCLC [3, 4]. The most successful immunotherapeutic drugs for NSCLC are immune checkpoint inhibitors (ICIs) targeting programmed death-1 (PD-1) and its ligand PD-L1 [5]. However, despite the significant progress, only a small fraction of patients attain durable clinical benefits [6, 7]. Approximately 70-75% of NSCLC patients are PD-L1 negative or low positive, necessitating combination strategies for effective treatment [8–10]. Even patients with high PD-L1 expression can develop resistance due to factors such as loss of neoantigens, defects in antigen presentation and interferon signaling, local immune dysfunction mediated by immune inhibitory molecules, and T cell exclusion [6, 11].

Enhancing the response rate and duration of anti-PD therapy is a major challenge in improving the efficacy of NSCLC immunotherapy [12]. One key approach to address the challenge is combining ICIs with other therapies to improve the tumor immune microenvironment (TIME) [13–15]. Numerous drugs have been tested in preclinical and clinical studies, but many have failed or are predicted to fail due to unclear mechanisms for improving the TIME [16, 17]. Beyond drugs, anti-tumor therapies based on physical agents—such as radiation, heat, light, and electricity—offer promising options for combination with immunotherapy, as they can directly modify the tumor microenvironment [18, 19].

Tumor-treating fields (TTFields) are an emerging therapeutic modality that has shown potential for combination with anti-PD immunotherapy. TTFields are intermediate-frequency (100–300 kHz) and low-intensity (1–3 V/cm) alternating electric fields. It could disrupt tumor cell mitosis by interfering with the alignment and movement of microtubules, leading to cell death and subsequent biochemical processes [20, 21]. At the same time, TTFields are a therapy with low side effects. Research has shown that TTFields do not affect the number of viable cells in non-cancerous BHK cell lines after replication inhibition nor in normal rat mesenteric and diaphragm tissues [20]. TTFields therapy has been approved for the treatment of glioblastoma multiforme (GBM) and malignant pleural mesothelioma (MPM) [22–24]. A phase 3 clinical trial (NCT02973789, LUNAR) demonstrated the efficacy of TTFields combined with anti-PD immunotherapy in NSCLC [25], leading to FDA approval of the combination therapy in October 2024. However, when the LUNAR study was launched in 2015, anti-PD drugs had not yet become the standard of care (SOC) for the treatment of advanced NSCLC. As a result, there was very limited preclinical research on the combination of TTFields and anti-PD immunotherapy, and little was known about its mechanism of action (MOA). While two proof-of-concept studies suggested a synergistic effect in cells and mice and indicated potential MOA related to immunology [26, 27], systematic preclinical investigation of the mechanism and impact of the combination therapy on the TIME is still lacking. Since TTFields therapy is mainly performed on the local tumor area, the mechanism by which TTFields improve the tumor microenvironment in combination with anti-PD therapy to achieve anti-tumor effects remains unclear. In-depth research on the mechanism of TTFields combined with anti-PD therapy for NSCLC will provide clues for identifying effective biomarkers to monitor the efficacy of the combined therapy.

In this study, we aim to uncover the MOA of TTFields therapy combined with anti-PD immunotherapy in NSCLC from the aspect of improving the TIME. Using a mouse model of NSCLC, we evaluated the efficacy of TTFields therapy with anti-PD-1 and anti-PD-L1 immunotherapies and compared them with anti-PD therapies alone. Inspired by RNA-seq analysis of tumor tissues, changes in the TIME after treatment were identified, including T cell infiltration, immunogenic cell death (ICD), and the activation of chemokine signaling pathways. Based on these findings, we conducted an investigation into these factors. This study provides deep insights of the MOA of TTFields therapy can be effectively integrated with anti-PD immunotherapy to improve treatment outcomes for NSCLC.

## Results

### TTFields therapy improved the efficacy of anti-PD-1 and anti-PD-L1 immunotherapy in the NSCLC mouse model

**Fig. 1:**
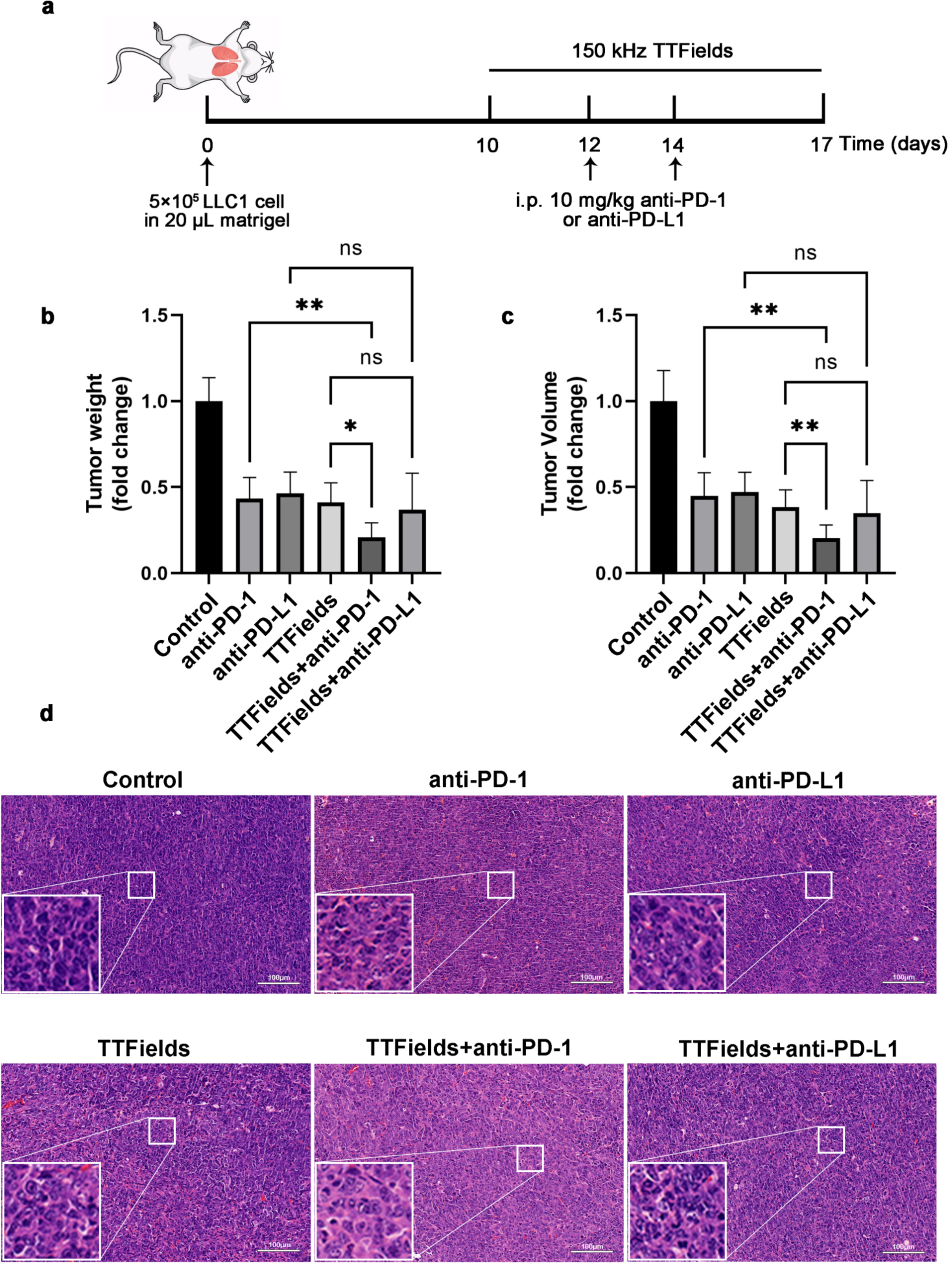
TTFields combined with anti-PD-1 enhances the antitumor effect of anti-PD-1 in vivo. (**a**) Schematic of the schedule of LLC1 tumor-bearing mouse model and treatment; (**b**) Tumor weight of control and every treatment group, *n* = 10/group; (**c**) Tumor volume of control and every treatment group, *n* = 10/group; (**d**) Representative H&E staining images of tumor tissue sections of every group (200×, Scale bar: 100 μm) (*p < 0.05, **p < 0.01, n.s. *p* ≥ 0.05)

The synergistic effects of TTFields therapy with anti-PD therapies were evaluated and compared with anti-PD therapies alone on a tumor-bearing mouse model of NSCLC. The NSCLC mouse model was constructed by in situ injecting LLC1 cells into the lung of the C57BL/6 mouse. The tumor-bearing mice were treated with anti-PD-1 antibody alone, anti-PD-L1 antibody alone, TTFields alone, TTFields with anti-PD-1 antibody, and TTFields with anti-PD-L1 antibody to evaluate and compare the anti-tumor efficacy of each treatment (Fig. 1a). H&E staining was used to evaluate the safety of TTFields therapy in major organs of healthy mice (Supplementary Fig. 1). Treatment with combination therapy or TTFields alone treatment had no effect on lung tissue without tumors (Supplementary Fig. 2), indicating that TTFields did not cause injury to major organs and was safe for normal tissues. However, TTFields increased white blood cells and neutrophils while decreasing lymphocyte numbers in the peripheral blood of wild-type mice (Supplementary Fig. 3), suggesting inflammation in both the Sham and TTFields groups. After treatment for 7 days, the tumor weight and volume of each mouse were measured (Fig. 1b and c). All the treatment groups demonstrated significant efficacy compared to the control group without treatment. The combination treatment with TTFields and anti-PD-1 significantly reduced the weight and volume of tumors in mice, compared to anti-PD-1 or TTFields treatment alone (*p* < 0.05). The combination treatment with TTFields and anti-PD-L1 could also reduce the tumor weight and volume compared to anti-PD-L1 or TTFields treatment alone, but the difference was not statistically significant (*p* > 0.05). It might indicate a different mechanism in the synergistic effects of TTFields therapy with anti-PD-1 and anti-PD-L1 immunotherapy.

Pathological examination of tumor tissue after treatment revealed that tumor nuclei in the control group without treatment were large and deeply stained, the nucleocytoplasmic ratio of tumor cells decreased, and nuclear staining became lighter in TTFields and anti-PD-1 treatment group, compared to control group (Fig. 1d). This suggests the cessation of DNA transcription and synthesis in tumor cells, indicating the occurrence of cell necrosis. Particularly, the TTFields and anti-PD-1 treatment group exhibited the most pronounced histological alterations (Fig. 1d). These data indicated that TTFields therapy and anti-PD immunotherapy had synergistic effects, and that TTFields therapy could enhance the efficacy of anti-PD immunotherapy.

### Combination therapy with TTFields and anti-PD changed tumor immune microenvironment

**Fig. 2:**
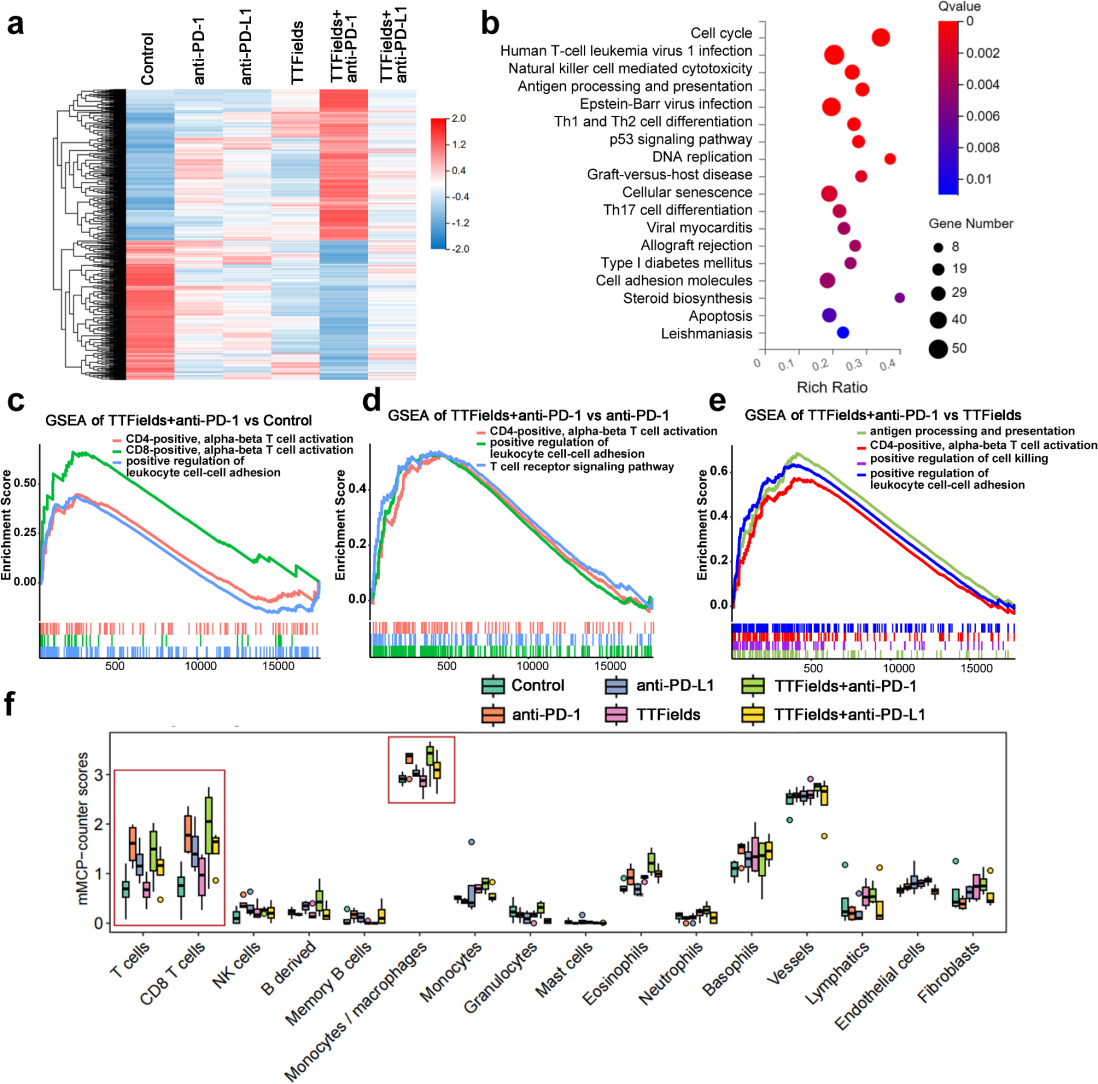
RNA-seq to explore changes in the immune microenvironment of LLC1 tumors after different treatments. (**a**) Heatmap showed the expression levels of DEGs with upregulated and downregulated expression among each group. Filled colors from blue to red represent expression levels from low to high; (**b**) Dot plot showed the enriched KEGG terms for upregulated DEGs of TTFields + anti-PD-1 vs. control group; (**c**-**e**) GSEA plots of DEGs signaling pathways in the TTFields + anti-PD-1 vs. control group, TTFields + anti-PD-1 vs. anti-PD-1 group, TTFields + anti-PD-1 vs. TTFields group (FDR < 0.05); (**f**) The box diagram showed the mMCP − counter scores of immune-infiltrating cells in each group

The TIME could affect the efficacy of anti-PD therapy [28]. To find out whether the combination therapy with TTFields and anti-PD could change the TIME, RNA-seq of tumor tissues was performed to compare the difference among various therapies at the transcriptional level. Compared to the control group, the number of differentially expressed genes in the groups treated with TTFields and anti-PD-1, TTFields and anti-PD-L1, anti-PD-1 alone, anti-PD-L1 alone, and TTFields alone, were 1745, 40, 241, 26, and 238, respectively (Fig. 2a). The most different genes were enriched in the TTFields and anti-PD-L1 treatment group. KEGG analysis showed that the upregulated genes after treatment with TTFields and anti-PD-1 could activate the classical cell cycle pathways and immune system pathways involving natural killer cell mediated cytotoxicity, antigen processing and presentation, cell differentiation of Th1, Th2, Th17 cells, etc. (Fig. 2b). Gene set enrichment analysis (GSEA) showed that regulated genes were significantly enriched in CD4^+^ alpha beta T cell differentiation, CD8^+^ alpha beta T cell activation, and positive regulation of leukocyte cell-cell adhesion after treatment with TTFields and anti-PD-1 (Fig. 2c). CD4^+^ alpha beta T cell differentiation, positive regulation of leukocyte cell-cell adhesion, and T cell receptor signaling pathway were more enriched in TTFields and anti-PD-1 treatment group than in the anti-PD-1 treatment group (Fig. 2d). Antigen processing and presentation, CD4^+^ alpha beta T cell differentiation, positive regulation of leukocyte cell-cell adhesion, and positive regulation of cell killing were more enriched in TTFields and anti-PD-1 treatment group than in the TTFields treatment (Fig. 2e). Murine microenvironment cell population counter (mMCP-counter) was used to evaluate the proportion of immune cells in mouse tissue samples from transcriptomic data. Results showed that the proportion of T cells, CD8^+^ T cells, monocytes, and macrophages was higher in TTFields and anti-PD-1 treatment group than in other treatment groups (Fig. 2f). Above all, RNA-seq showed TTFields and anti-PD-1 treatment could change the TIME.

### TTFields enhanced intratumor T cell infiltration

**Fig. 3:**
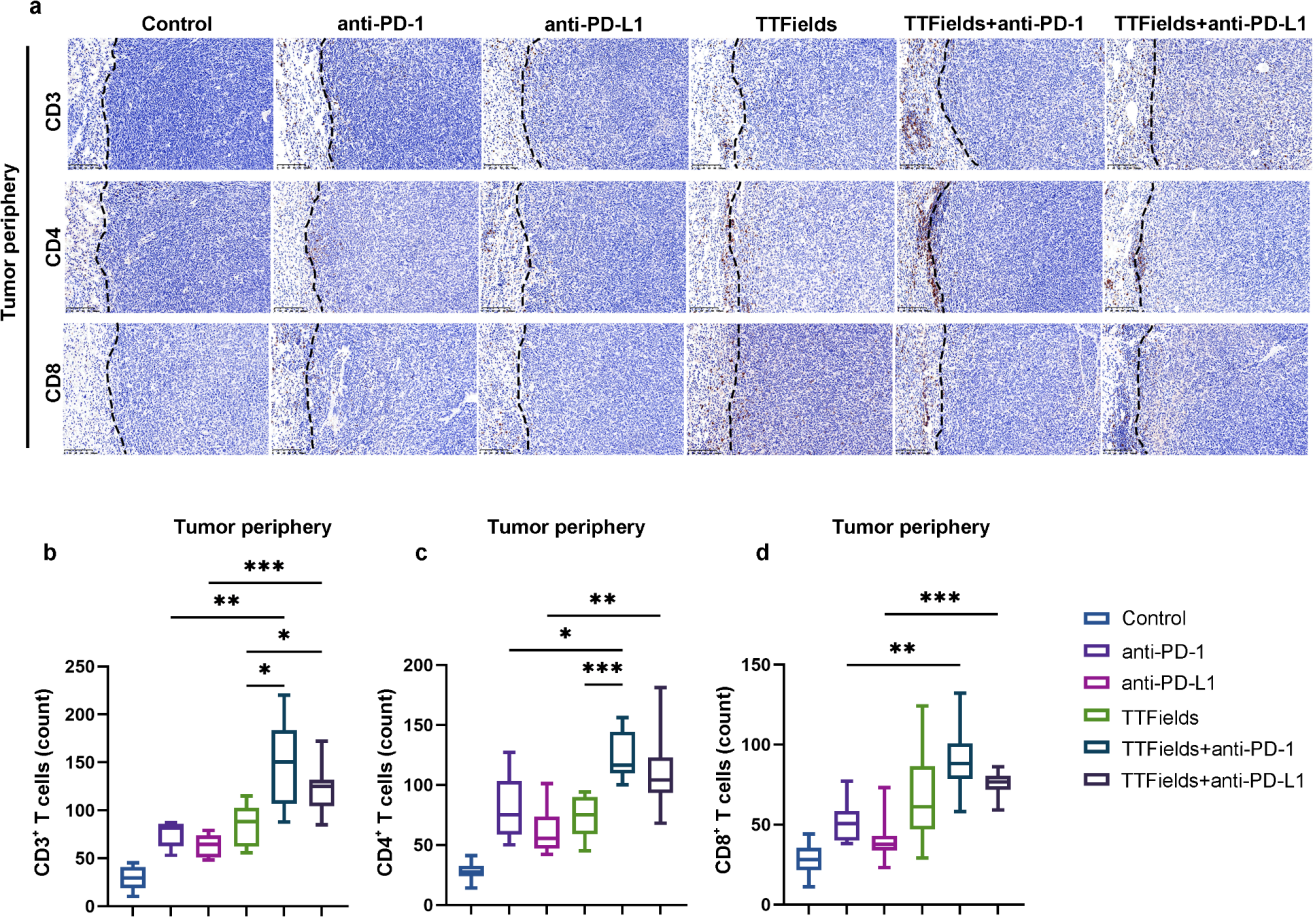
Immunohistochemistry (IHC) analysis of CD3^+^ T cells, CD4^+^ T cells and CD8^+^ T cells in LLC1 tumor periphery. (**a**) IHC images of CD3^+^ T cells, CD4^+^ T cells and CD8^+^ T cells in the tumor periphery of every group; (**b**-**d**) Quantitative analysis of (**b**) CD3^+^ T cells, (**c**) CD4^+^ T cells and (d) CD8^+^ T cells in the tumor periphery of every group. The scale bar is 100 μm (*n* = 10/group, *p < 0.05, **p < 0.01)

**Fig. 4:**
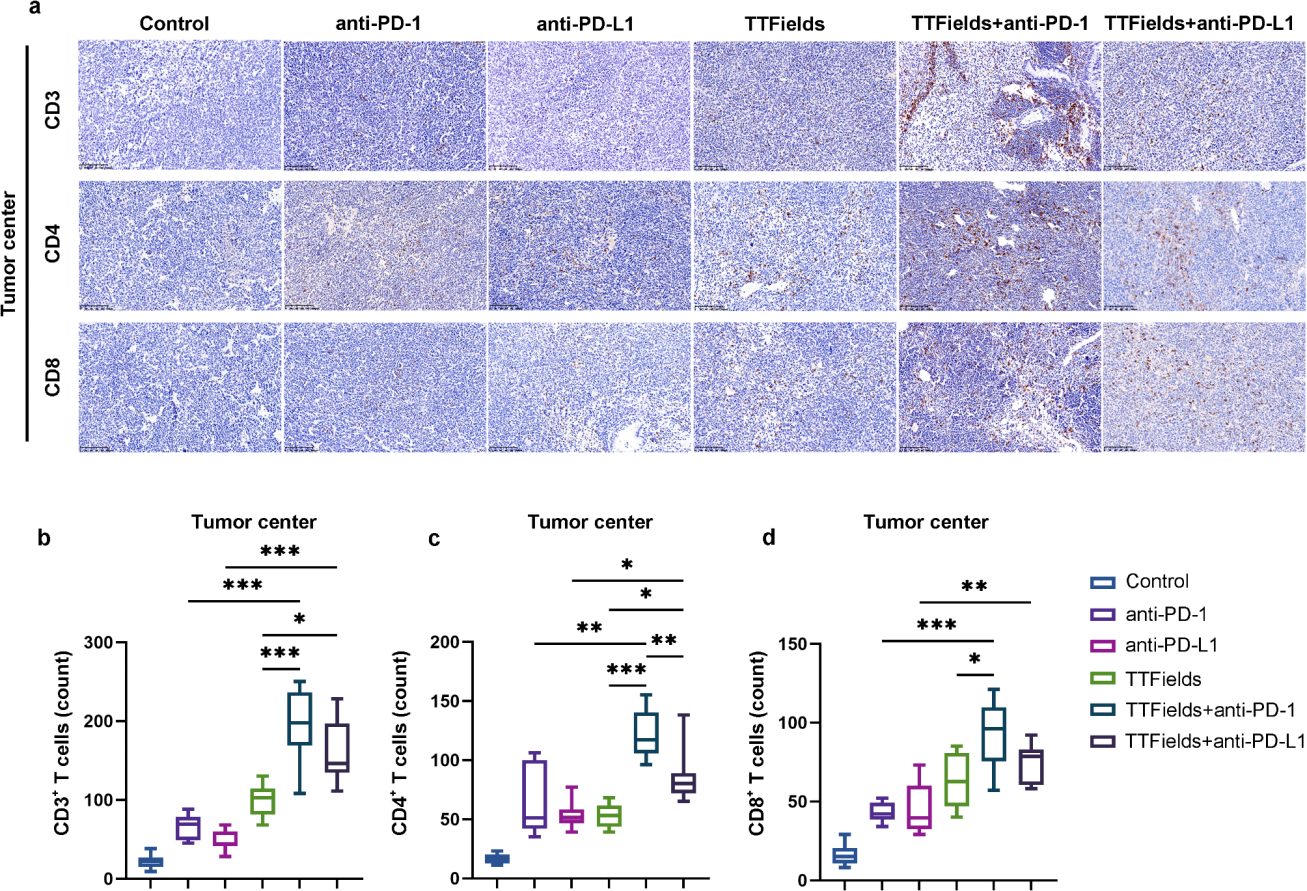
Immunohistochemistry (IHC) analysis of CD3^+^ T cells, CD4^+^ T cells and CD8^+^ T cells in LLC1 tumor center. (**a**) IHC images of CD3^+^ T cells, CD4^+^ T cells and CD8^+^ T cells in the tumor center of every group; (**b**-**d**) Quantitative analysis of (**b**) CD3^+^ T cells, (**c**) CD4^+^ T cells and (d) CD8^+^ T cells in the tumor center of every group. The scale bar is 100 μ (*n* = 10/group, *p < 0.05, **p < 0.01)

T cell infiltration is a key factor of TIME and a direct clinical indicator for predicating the efficacy of anti-PD therapies [6, 11, 29]. To test whether TTFields could enhance T cell infiltration, the distribution and quantification of CD4^+^ and CD8^+^ T cells were analyzed in tumor tissues via immunohistochemistry. The number of T cells in the periphery and center of tumor tissues was counted to assess the degree of T cell infiltration (Figs. 3a and 4a and Supplementary Fig. 4). The combination treatment with TTFields and anti-PD-1 facilitated more infiltration of CD3^+^, CD4^+^ and CD8^+^ T cells into both the periphery and center of the tumor tissue, than anti-PD-1 treatment alone (Figs. 3b-d and 4b-d). The combination treatment with TTFields and anti-PD-L1 could also facilitate more T cell infiltration than anti-PD-L1 treatment alone. Furthermore, the combination treatment with TTFields and anti-PD-L1 recruited fewer CD4^+^ T cells into the tumor center than the combination treatment with TTFields and anti-PD-1 (Fig. 4c). These data provided direct evidence that TTFields could improve the intratumor T cell infiltration in TIME of NSCLC, thus resulting in the improved efficacy of anti-PD therapy.

### Combination therapy with TTFields and anti-PD induced ICD of tumor cells

It has reported that ICD can establish a dialogue between dying cells and immune cells to activate an adaptive immune response and enhance the infiltration of T cells into tumor tissue [30]. To explore whether the combination therapy with TTFields and anti-PD induced ICD of tumor cells, we examined ICD-related biomarkers in vitro and in vivo assays.

**Fig. 5:**
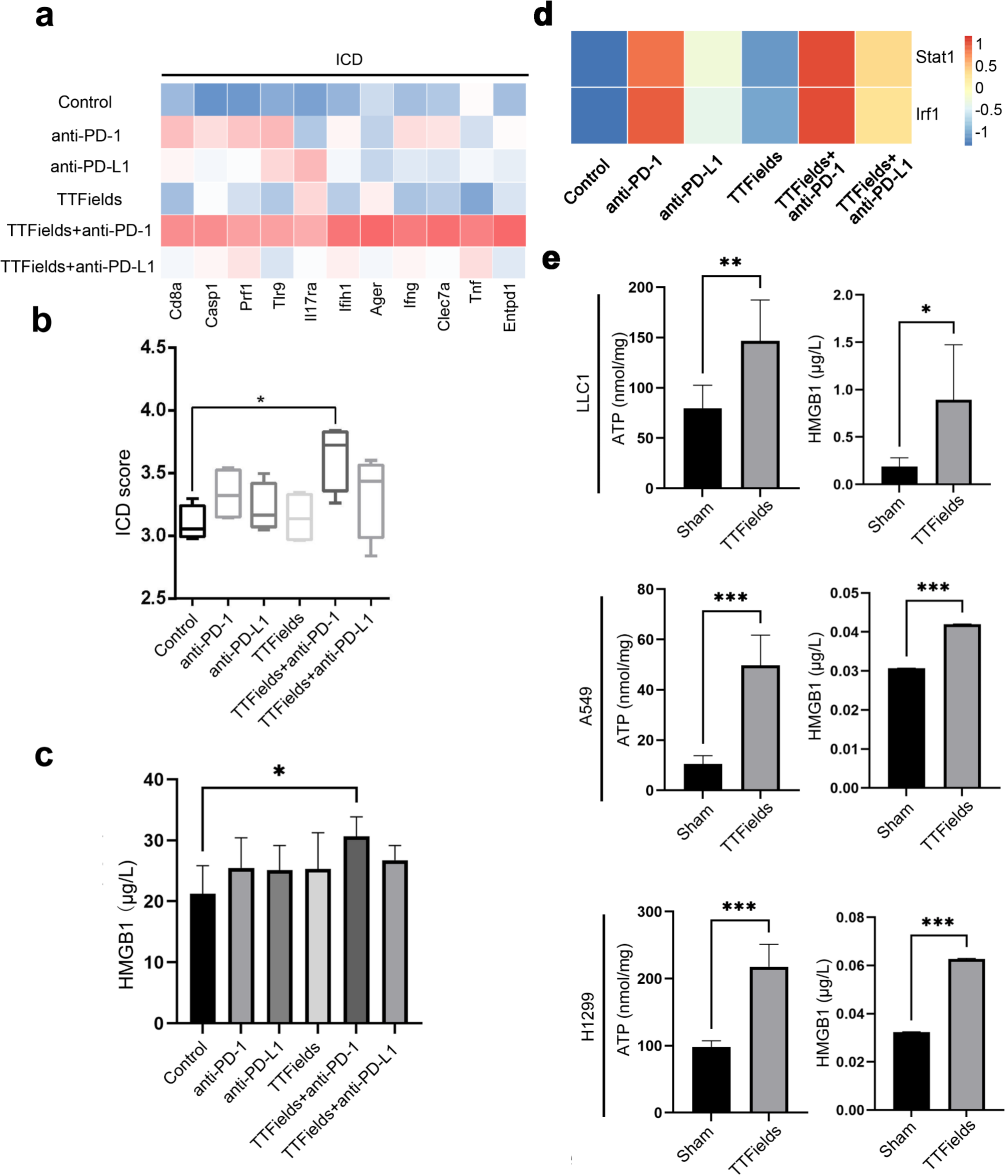
TTFields induces ICD in vitro and in vivo. (**a**) Heatmap showed fragments per kilobase million (FPKM) quantification of representative genes in the ICD pathway. Filled colors from blue to red represent expression levels from low to high; (**b**) The ICD score in each group (*p < 0.05); (**c**) Secreted HMGB1 in LLC1 tumor with anti-PD-1, anti-PDL1, TTFields therapy alone or TTFields plus anti-PD-1/PD-L1 was detected by ELISA (*n* = 6, *p < 0.05). (**d**) Heatmap showed fragments per kilobase million (FPKM) quantification of *STAT1* and *IRF1*. (**e**) TTFields induces ICD in A549, H1299 and LLC1. ATP secretion was detected by chemiluminescence assay. Secreted HMGB1 in the culture supernatant of TTFields treatment or not were detected by ELISA (*n* = 6, *p < 0.05, **p < 0.01, ***p < 0.001)

Various ICD associated genes were expressed at different levels in the tumor of each treatment group (Fig. 5a). The TTFields and anti-PD-1 treatment group exhibited the highest expression level of ICD associated genes including *CD8a*,* Casp1*,* TLR9*,* IL17Ra*,* IFN-g*, and *TNF.* The ssGSEA algorithm was used to analyze the ICD score of each group. The TTFields and anti-PD-1 treatment group showed the highest score (Fig. 5b). The HMGB1, an ICD biomarker, was tested in each group. The TTFields and anti-PD-1 treatment group showed higher HMGB1 levels than anti-PD-1 treatment alone and TTFields treatment alone, although the differences were not statistically significant (Fig. 5c). Since the inflammatory response is the additional effect induced by ICD [31], the release of multiple cytokines including IFN-γ, IL-1β, IL-6, IL-18 and TNF-α was examined. The TTFields and anti-PD-1 treatment group induced more release of IL-1β and IL-18 in tumor than anti-PD-1 treatment alone (Supplementary Fig. 5). Transcription factors *Stat1* and *Irf1* were also more enriched in combination therapy with TTFields and anti-PD-1 than anti-PD-1 treatment alone or TTFields treatment alone (Fig. 5d). Owing to the expressions of *Stat1* and *Irf1* are associated with the activation of the IFN-γ signaling pathway and the NF-κB pathway, respectively. these findings suggest that combination therapy with TTFields and anti-PD1 activates ICD in tumor cells, which, in turn, activates the IFN-γ signaling pathway and the NF-κB pathway to release multiple cytokines.

As the detection of biomarkers of ICD might be affected by the complicated tumor microenvironment and the ICD was most likely to be caused by TTFields, we further investigated whether TTFields could induce ICD in vitro. The mouse NSCLC cell line LLC1, and two human NSCLC cell line A549 and H1299 were tested. It was confirmed that TTFields could reduce cell survival rate and inhibit cell clonogenesis and migration (Supplementary Fig. 6) as previously reported. After TTFields treatment in vitro for 72 h, The ATP and HMGB1 were significantly upregulated in LLC1 cells, A549 cells, and H1299 cells. (Fig. 5e). These data indicated that TTFields could induce ICD not only in the NSCLC mouse model but also in human NSCLC. The release of cytokines (IFN-γ, IL-1β, IL-6, IL-18 and TNF-α) related to immune response was upregulated after TTFields treatment in vitro (Supplementary Fig. 7a-c). These results suggested that TTFields were able to trigger ICD and improve the TIME in vitro.

### Combination therapy with TTFields and anti-PD activated CCL2/8-CCR2 and CXCL9/10-CXCR3 axis

**Fig. 6:**
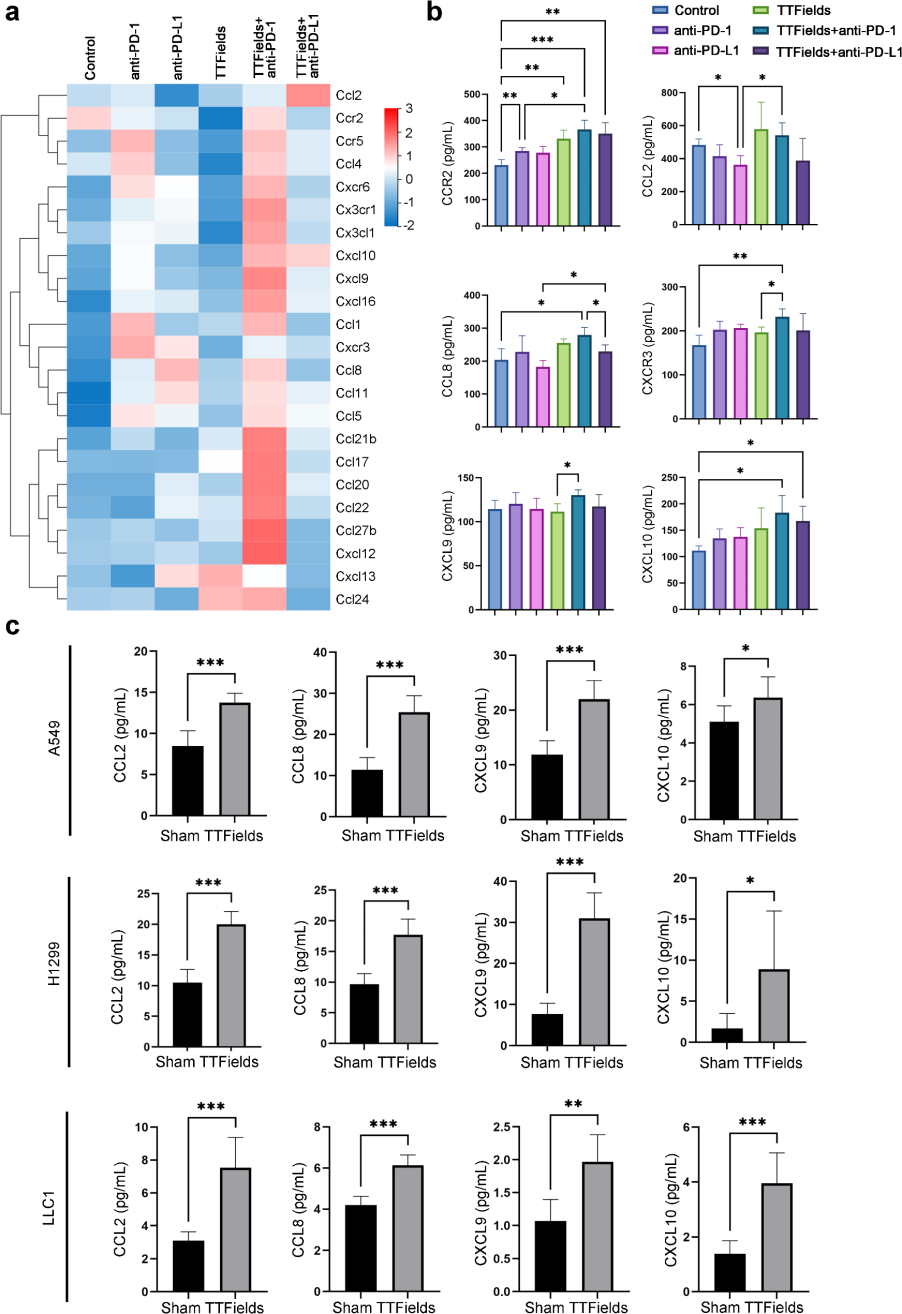
TTFields combined with anti-PD-1 further activates immune-related chemokine pathways. (**a**) Heatmap of genes that were related to immune-related chemokine pathways (Based on fragments per kilobase million (FPKM) quantification). Filled colors from blue to red represent expression levels from low to high; (**b**) Expression levels of chemokines and their receptors, including CCR2, CCL2, CCL8, CXCR3, CXCL9 and CXCL10, from tumor tissue of anti-PD-1, anti-PDL1, TTFields therapy alone or TTFields plus anti-PD-1/PDL1 therapy group were detected by ELISA (*n* = 6, *p < 0.05, **p < 0.01, ***p < 0.001); (**c**) TTFields induces chemokine secretion in A549, H1299 and LLC1. Secreted CCL2, CCL8, CXCR3, CXCL9 and CXCL10 in the culture supernatant of tumor cells with TTFields treatment or not were detected by ELISA (*n* = 6, *p < 0.05, **p < 0.01, ***p < 0.001)

The occurrence of ICD can induce the production of chemokines, which contribute to improving the TIME. We analyzed chemokine-related gene sets and found that many chemokines and their receptors were activated after TTFields and anti-PD-1 treatment (Fig. 6a). Increased chemokines could promote an immunotherapeutic response by recruiting immune cells with antitumor activity into the tumor, thereby killing tumor cells [32–34]. Among them, the CXCL9/CXCL10-CXCR3 axis regulated the differentiation of naive T cells to T helper 1 (Th1) cells. Migration of immune cells to tumor sites, meanwhile CCL2/8-CCR2 axis had the function of chemotactic mononuclear/macrophage cells and CD8^+^ T cells [35–37]. In mice lung tumor tissues, both chemokines and their receptors of these two signaling axis were significantly upregulated after TTFields and anti-PD-1 treatment, while TTFields plus anti-PD-L1 treatment failed to activate CCL8 and CXCR3 (Fig. 6b). TTFields and anti-PD-1 treatment triggered higher CCR2 expression than anti-PD-1 treatment only (Fig. 6b). TTFields and anti-PD-1 treatment induced greater expression of CXCR3 and CXCL9 than TTFields treatment alone (Fig. 6b), suggesting the TTFields and anti-PD-1 treatment trigger more chemokine axes to recruit T cells infiltrating and alter the TIME.

The protein expressions of CCL2, CCL8, CXCL9 and CXCL10 were also upregulated in A549, H1299 and LLC1 cells after treatment with TTFields in vitro for 72 h (Fig. 6c). These data suggested that TTFields treatment on tumor cells promoted the production of chemokines for recruiting immune cells. Then we verified the protein expressions of CCL21, CCL24, CXCL13, CCR5-CCL5 and CXCR6-CXCL16 by ELISA, which were involved in the regulation of granulocytes, NK cells, T cells, macrophages, and other immune cells. These chemokines (CCL21, CCL24, CXCL13, CCL5 and CXCL16) were activated and released after treatment with TTFields in vitro (Supplementary Fig. 8), while the upregulations of CCR5, CCL21 and CXCR6 were not statistically significant in vivo (Supplementary Fig. 9). Above all, these results indicated that the CCL2/8-CCR2 axis and CXCL9/10-CXCR3 axis were involved in the anti-tumor immunity of the TTFields and anti-PD-1 treatment.

Moreover, TRRUST database analysis showed that *Stat1* and *Irf1* are transcription factors for the chemokines CXCL9/10 and CCL2 (supplementary Fig. 10). In summary, TTFields-induced ICD actives *Stat1* and *Irf1*, which promote the accumulation of CD8^+^ T cells and CD4^+^ T cells via the CCL2/8-CCR2 axis and CXCL9/10-CXCR3 axis.

## Discussion

In this study, we find that the combination therapy with TTFields and anti-PD-1 performs better than the combination therapy with TTFields and anti-PD-L1 in the efficacy and improvement of TIME. The effective advantage of combining therapy with TTFields and anti-PD-1 is that it promotes the infiltration of a large number of CD8^+^ T and CD4^+^ T cells into the tumor tissue, thereby improving the tumor microenvironment. This combination therapy induces ICD in tumor cells, which activates the IFN-γ and NF-κB signaling pathways. These pathways further promote the expression of CXCL9/10 and CCL2 through the IFNγ/STAT1/IRF-1 signaling pathway [38–40]. Therefore, the combination therapy with TTFields and anti-PD-1 activates STAT1 and IRF1 through ICD in tumor tissue, enhancing the CCL2/8-CCR2 axis and CXCL9/10-CXCR3 axis, which recruits CD8^+^ T and CD4^+^ T cells to infiltrate and kill tumor cells. Although there have been reported about the combination therapy with TTFields and anti-PD in NSCLC [26, 27]. The main aim of the two studies was to evaluate the efficacy of TTFields in combination with a PD-1 inhibitor, a PD-1 + CTLA-4 inhibitor, or a PD-L1 inhibitor. Flow cytometry analysis of immune cells was mainly conducted in both studies. Most of the data serve as proof-of-principle evidence. The MOA of the combination therapy was not comprehensively explored. Our study is the first to reveal the mechanism by which combination therapy improves the tumor microenvironment and to compare the effects of different combination therapy regimens. CCL2/8-CCR2 and CXCL9/10-CXCR3 could serve as potential biomarkers for predicting patient response to TTFields and anti-PD therapy in NSCLC. Monitoring chemokine concentrations in peripheral blood and changes in the ratio of CD4^+^ T and CD8^+^ T cells could indicate an improvement in the immune status of cancer patients, which may be useful for evaluating the efficacy of tumor treatment in the future. However, extensive clinical quantitative research is still needed to assess the effects of combination therapy at different stages of non-small cell lung cancer, which would help personalize treatment strategies.

In previous studies, TTFields were found to promote ICD in GBM and colorectal cancer [26, 41]. The effect of TTFields therapy on ICD was not systematically studied in NSCLC. In this study, we systemically evaluated the effect of TTFields on ICD of tumor cells in vitro and in vivo. Its mechanism was investigated as well. The effect of TTFields on chemokine production from NSCLC tumor cells has not been studied in previous studies. In this study, both transcriptional and protein analyses revealed upregulation of CCL2/8-CCR2, as well as the CXCL9/10-CXCR3 axis after TTFields and anti-PD-1 treatment, while the regulation after TTFields and anti-PD-L1 treatment was not significant.

The phase 3 clinical trial evaluating the efficacy and safety of the combination therapy of TTFields and an anti-PD antibody for second-line treatment of NSCLC (LUNAR, NCT02973789) showed that the addition of TTFields therapy significantly improved overall survival compared with an immune checkpoint inhibitor alone. The median overall survival was 18.5 months (95% CI: 10.6–30.3) in the combination therapy group versus 10.8 months (95% CI: 8.2–18.4) in the immune checkpoint inhibitor group, with a hazard ratio (HR) of 0.63 (95% CI: 0.41–0.96). However, the LUNAR study was initiated without thorough preclinical studies due to the rapid changes in the standard of care (SOC) for the treatment of advanced NSCLC. The LUNAR trial was previously designed to evaluate the efficacy and safety of TTFields with SOC for second-line treatment of NSCLC in 2015. Chemotherapy was the only SOC in 2015. But since the end of 2015, anti-PD drugs have been approved and joined the SOC for NSCLC rapidly. The clinical trial was then modified to evaluate the efficacy and safety of TTFields with chemotherapy or anti-PD immunotherapy. That is why insufficient preclinical studies were conducted before the phase 3 clinical trial. Currently, the anti-PD immunotherapy has been moved to the first-line treatment for advanced NSCLC, together with chemotherapy. However, the mechanism of TTFields combined anti-PD therapy has not been elucidated yet. Our explanation of the mechanism of such combined therapy will provide theoretical basis for TTFields combined anti-PD therapy application and new ideas for precision treatment. Compared to other studies or treatment modalities [26, 27], although we have studied the mechanism of TTFields therapy, several questions remain for further investigation. For example, the difference MOA between the efficacy of TTFields therapy with anti-PD-1 and anti-PD-L1 immunotherapy have not been fully elucidated. TTFields can directly affect the local tumor and modify the tumor microenvironment. The anti-PD-1 and anti-PD-L1 antibodies are likely to elicit different responses to changes in the TIME. The components of the PD-1/PD-L1 axis may engage distinct immune cells or pathways in the context of TTFields [42, 43]. Therefore, the synergistic mechanism between TTFields and anti-PD therapy still requires further exploration. Additionally, the impact of TTFields on immune-related adverse events (irAEs) remains unclear [44–46], despite evidence suggesting that localized TTFields may paradoxically mitigate systemic inflammation. Developing specialized treatment plans for patients at different stages and exploring the relationship between various electric field conditions, immune states, and the generation of irAEs will be benefit for identifying the optimal combination strategy for anti-tumor therapy in the future. Since only one combination dosage was studied in this work, factors such as the intensity, treatment duration, and timing of TTFields, as well as the dosage and frequency of anti-PD antibodies, should be further investigated. Additionally, the heterogeneity of tumor subgroups in NSCLC and the varying tumor microenvironments at different stages may lead to different responses to TTFields [47–52]. Thus, selecting the optimal timing, duration, and intensity of TTFields therapy in combination with anti-PD immunotherapy to maximize efficacy requires further study. Additionally, although TTFields have minimal impact on the mitosis of normal cells and do not alter the permeability of their cell membranes [20, 53], the effects of TTFields on normal cells is undoubtedly valuable for further studying to explore an effective and safe anti-tumor treatment strategy. So the suitable combinations therapy of TTFields and anti-PD will be further screened to achieve safe and effective treatment. Moreover, a deeper study of the mechanisms underlying different TTFields combination therapies will provide a theoretical basis for elucidating the efficacy of anti-tumor therapy.

## Methods

### Design of animal experiments

8- to 10-week-old male C57BL/6 mice were purchased from Cavens Laboratories (Changzhou, China), and in vivo experiments were performed according to the guidelines of the International Guidelines for Animal Research and approved by the Ethics Committee of Shandong First Medical University (W202311160311). Mice were fed and experimented under specific pathogen-free grade conditions. After anesthetizing the mouse with isoflurane, make a small incision approximately 1 cm above the lower edge of the left rib cage (between the fourth and fifth rib arches). Gradually cut through the underlying subcutaneous and muscle tissue to expose the lung. The pink lung tissue should be visible through the pleura and will expand and contract with the mouse’s breathing. Inject 20 μl of Matrigel containing 5 × 10⁵ LLC1 cells slowly along the incision, targeting the left lung. The needle should be inserted to a depth of approximately 3 mm. After completing the injection, hold the needle in place for 5 s, then slowly withdraw it with gentle rotational movements. Ten days after tumor cell injection, the mice (*n* = 10) were randomly treated with anti-PD-1 antibody (*i.p.*, 10 mg/kg, on day 12 and day 14), anti-PD-L1 (*i.p.*, 10 mg/kg, on day 12 and day 14), TTFields (150 kHz, for 7 days), a combination of TTFields and anti-PD-1 antibody, or a combination of TTFields and anti-PD-L1 antibody. Mice in the control group were not treated. Subsequently, the mice were euthanized by cervical dislocation on day 17, and the tumors were excised for further analysis. tumor size (length×width^2^/2) and tumor weight were recorded, and differences among groups were compared by fold change (fold change of every group = the tumor size or weight of treatment group / the mean values of the tumor size or weight of control group).

The TTFields device generates a 150 kHz sine wave electric field that is output and loaded onto two pairs of electrode pads attached perpendicularly to each other on either side of the animal’s trunk. The electrode pads were attached to the skin of the mouse with hydrogel, and the electrode pads were replaced every 2–3 days to ensure tight fit and continued treatment. The current was maintained at approximately 60 mA by the current monitoring software to prevent scalding of the mouse’s skin due to the high temperature.

### Hematoxylin-eosin (H&E) staining and immunohistochemistry (IHC)

Isolated lung tissues and tumors were fixed with 4% paraformaldehyde for 48 h, dehydrated, and embedded in paraffin wax for further experiments. Hematoxylin-eosin (H&E) staining, and various IHC assays were performed according to the manufacturer’s instructions. The primary antibodies used in the experiments were as follows: anti-CD3 (1:150, ab16669, Abcam, ), anti-CD4 (1:200, 25229, CST), and anti-CD8 (1:500, ab237709, Abcam). Bright-field images were captured using a digital pathology section scanner (KFBIO, China). For CD3^+^, CD4^+^ and CD8^+^ T cell analysis, tumor center and tumor periphery T cells were counted separately, 10 random fields in each group.

### RNA‑seq assay

Mice lung tumor tissues were sent to BGI Genomics (The Beijing Genomics Institute) for RNA isolation, library construction and sequencing, with four biological replicates in each group. An online software (http://report.bgi.com) developed by BGI was utilized for data analysis and image plotting, including clustering heatmap and KEGG. Briefly, differential expression analysis was performed on each using the DESeq2 R package, and hierarchical clustering analysis was performed using the R package pheatmap. Gene differential expression was considered significant when Qvalue (Adjusted *p*-value) was less than 0.05. KEGG enrichment analysis was performed based on the differential genes, and *P*-values and false discovery rates (FDRs) were calculated using the phyper function in the R project. GSEA analysis was performed using the official GSEA software package (gsea-msigdb.org). Immune infiltration analysis referenced to the method reported by Florent Petitprez [54]. To evaluate the degree of ICD in each sample, Single sample gene set enrichment analysis (ssGSEA) was used to calculate ICD score by entering an ICD gene set [55, 56]. TRRUST database (https://www.grnpedia.org/trrust) was used to predict the transcription factors regulating the chemokines CXCL9/10 and CCL2.

### Enzyme-linked immunosorbent assays (ELISA)

For mice lung tumor tissues, 10% tissue homogenate was made with PBS, and after centrifugation, its supernatant was used to measure the HMGB1, IFN-γ, IL-1β, IL-6, IL-18, TNF-α, CCL2, CCL5, CCL8, CCL21, CCL24, CCR2, CCR5, CXCR3, CXCR6, CXCL9, CXCL10, CXCL13, and CXCL16 levels by enzyme-linked immunosorbent assay (ELISA) kits (MEIMIAN, China). For tumor cell lines, supernatants were obtained after 72 h TTFields (150 kHz, 1.81 ± 0.15 V/cm for A549, 1.76 ± 0.12 V/cm for H1299, 2.14 ± 0.20 V/cm for LLC1) to measure the secretion of HMGB1, IFN-γ, IL-1β, IL-6, IL-18, TNF-α, CCL2, CCL5, CCL8, CCL21, CCL24, CXCL9, CXCL10, CXCL13, CXCL16 by using ELISA kits (MEIMIAN, China). Absorbance at 450 nm was measured for each well according to the manufacturer’s instructions, and then chemokine concentrations were obtained by extrapolation from a standard curve. Standard curves were obtained by doubling the dilution of ELISA kit standards.

### Cell culture

The human non-small cell lung cancer cell lines A549, H1299 and the mouse non-small cell lung cancer cell line LLC1 with low PD-L1 expression were purchased from the Cell Bank of the Chinese Academy of Sciences (Shanghai, China) [57, 58]. The cells were grown in Dulbecco’s Eagle’s medium (DMEM, Gibco) containing 10% heat inactivated fetal bovine serum (FBS, Gibco) and 1% penicillin-streptomycin at 37℃ in 5% CO_2_.

### In vitro TTFields experiment system

TTFields were applied to the cells using the in vitro system. The device mainly consists of a tumor treating fields therapy instrument and a cell experimental tooling (Supplementary Fig. 11). The 150 kHz sinusoidal wave signal generated by the tumor treating fields therapeutic instrument was transmitted to two pairs of insulated electrodes composed of ceramic sheets. The two pairs of electrodes were inserted perpendicular to each other inside the culture dish. The commutation voltage generated by the tumor treating fields therapeutic instrument is loaded alternately on the two pairs of electrodes, thus switching the direction of the electric field every 1 s. The thermistor on the electrodes can record the temperature data of the culture fluid in real time and set a specific incubator environment temperature according to the voltage of TTFields, to control the temperature inside the culture fluid in the range of 36.5–37.5℃ during the experiment. The higher the output voltage of TTFields, the stronger the alternating electric field generated, and the lower the incubator set temperature. Sham group cells were placed in an TTFields device without applying TTFields.

### In vitro cytotoxicity assay

To detect the effect of electric field intensity on cytotoxicity, electric fields with field intensities of 1.0 ∼ 2.2 V/cm were applied to the cells at a frequency of 150 kHz for 72 h. Cytotoxicity was determined by cell counting using Scepter™ 3.0 (Merck, Germany).

### Clonogenicity

For colony formation assay, cells that had been applied TTFields for 72 h were placed into 6-well tissue culture plates (A549 400 cells/well, H1299 400 cells/well, LLC1 1000 cells/well) and cultured in DMEM medium containing 10% FBS for 10 to 15 days, and the medium was changed every 5 days. Then, colonies were fixed with 4% polyformaldehyde and stained with 1% crystal violet (Beyotime, China) in PBS for 10 min. Colony forming ability was analyzed by counting the number of stained colonies. Three replicates were performed for each group.

### Wound healing

Place the cells in a 12-well plate. When the cells grew to more than 90% confluence, a scratch wound was formed with a 200 μL pipette tip and rinsed three times with PBS to remove the cells that were scratched down. Subsequently, serum-free medium was added, initial time point pictures were taken, and the cells were placed in the incubator for electric field application. After a period, each group of cell crawls was removed for observation and photographed. The cell migration rate was calculated by comparing the area of the scratch at the initial scratch (0 h) and the scratch at the later observation point using ImageJ.

### ATP release

To detect ATP release, cell culture supernatants from sham or TTFields groups were collected and assayed according to the manufacturer’s instructions (Beyotime, China).

### Statistical analyses

The data are represented as mean ± SD. Statistical significance was determined by a 2-tailed Student’s t-test and a one-way analysis of variance (ANOVA) Friedman’s test. For one-way ANOVA, a homogeneity test of variance was first performed, and if the variance was uneven, a Brown-Forsythe and Welch ANOVA was used. GraphPad PRISM version 9.0 was used for the statistical analyses, and *p < 0.05 was considered as a statistically significant difference.

## Electronic supplementary material

Below is the link to the electronic supplementary material.

Supplementary Material 1

## Data Availability

The data that support the findings of this study are available from the corresponding author upon reasonable request. The RNA-Seq data was deposited in [GSA] repository (Accession code CRA021337, https://ngdc.cncb.ac.cn/gsa/browse/CRA021337).

## Abbreviations

ELISA: Enzyme-linked immunosorbent assays
FDRs: False discovery rates
GBM: Glioblastoma
GBM: Glioblastoma multiforme
GSEA: Gene set enrichment analysis
H&E: Hematoxylin-eosin
ICD: Immunogenic cell death
ICIs: Immune checkpoint inhibitors
irAEs: Immune-related adverse events
mMCP-counter: Murine microenvironment cell population counter
MOA: Mechanism of action
MPM: Malignant pleural mesothelioma
NSCLC: Non-small cell lung cancer
PD-1: Programmed death-1
SOC: Standard of care
ssGSEA: Single sample gene set enrichment analysis
TIME: Tumor immune microenvironment
TTFields: Tumor treating fields
