# Supplementary material for: Tumor treating fields enhance anti-PD therapy by improving CCL2/8 and CXCL9/CXCL10 expression through inducing immunogenic cell death in NSCLC models
Source: BMC Cancer. 2025 Mar 17;25:489. doi: 10.1186/s12885-025-13859-w (PMC11912744; doi:10.1186/s12885-025-13859-w)
Supplement: Supplementary file 1 — Supplementary Material 1 [file 12885_2025_13859_MOESM1_ESM.docx]

**Tumor Treating Fields enhance anti-PD therapy by improving CCL2/8 and CXCL9/CXCL10 expression through inducing immunogenic cell death in NSCLC models**

Wei Lin^1,2#*^, Yingying Wang^3#^, Minghao Li^1#^, Jingjing Feng^3^, Ying Yue^3^, Jing Yu^3^, Yanjiang Hu^4*^, Yuanzhen Suo^3,5*^

^1^Public Scientific Research Platform, School of Clinical and Basic Medicine, Shandong First Medical University & Shandong Academy of Medical Sciences, Jinan 250118, China

^2^Department of Critical-care Medicine, Shandong Provincial Hospital Affiliated to Shandong First Medical University, Jinan 250021, China

^3^Healthy Life Innovation Medical Technology Co., Ltd, Wuxi 214174, China

^4^Department of Thoracic Surgery, Liyang People's Hospital, Liyang 213300, Jiangsu, China.

^5^Liangzhu Laboratory, Zhejiang University, Hangzhou 310058, China

*Correspondence to: Yuanzhen Suo (suoyuanzhen@zju.edu.cn), Wei Lin (linw1978@163.com), and Yanjiang Hu (13815096789@163.com)

#These authors contributed equally to this work

**Supplementary Figure 1**

**
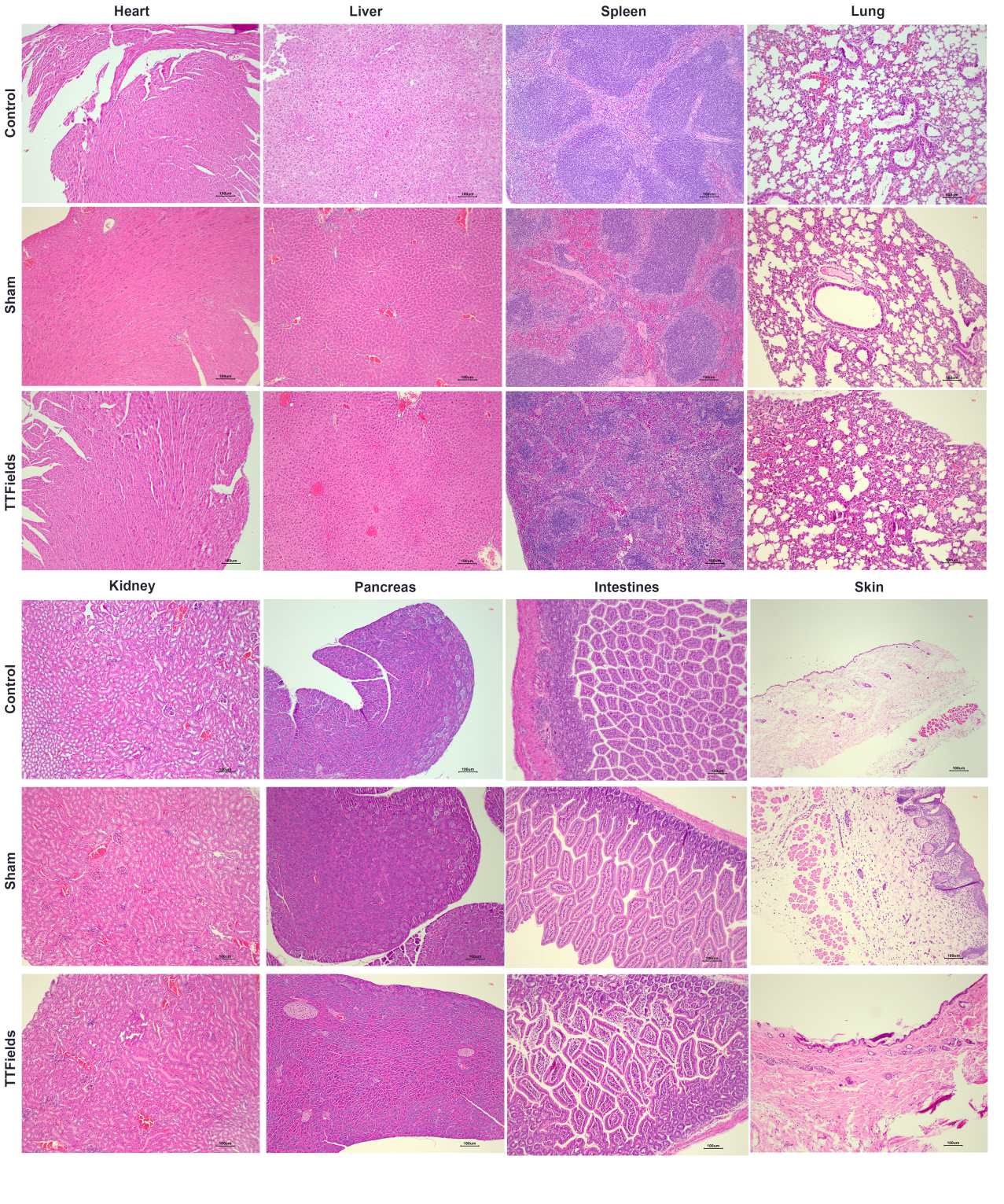
**

**Supplementary Figure 1 Representative H&E staining images of major organ tissue sections of healthy mice** (100×, Scale bar: 100 μm)**.**

**Supplementary Figure 2**

**
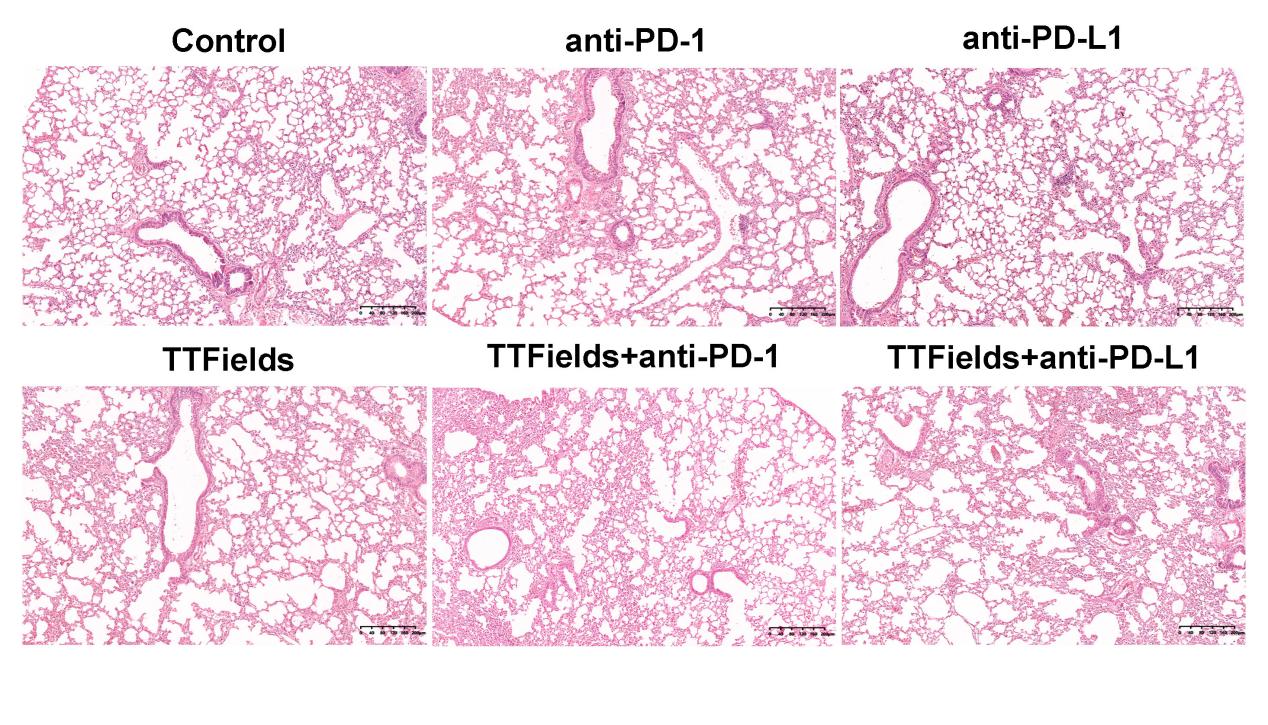
**

**Supplementary Figure 2 Representative H&E staining images of lung tissue sections of mice** **after treatment** (100×, Scale bar: 200 μm)**.**

**Supplementary Figure 3**

**
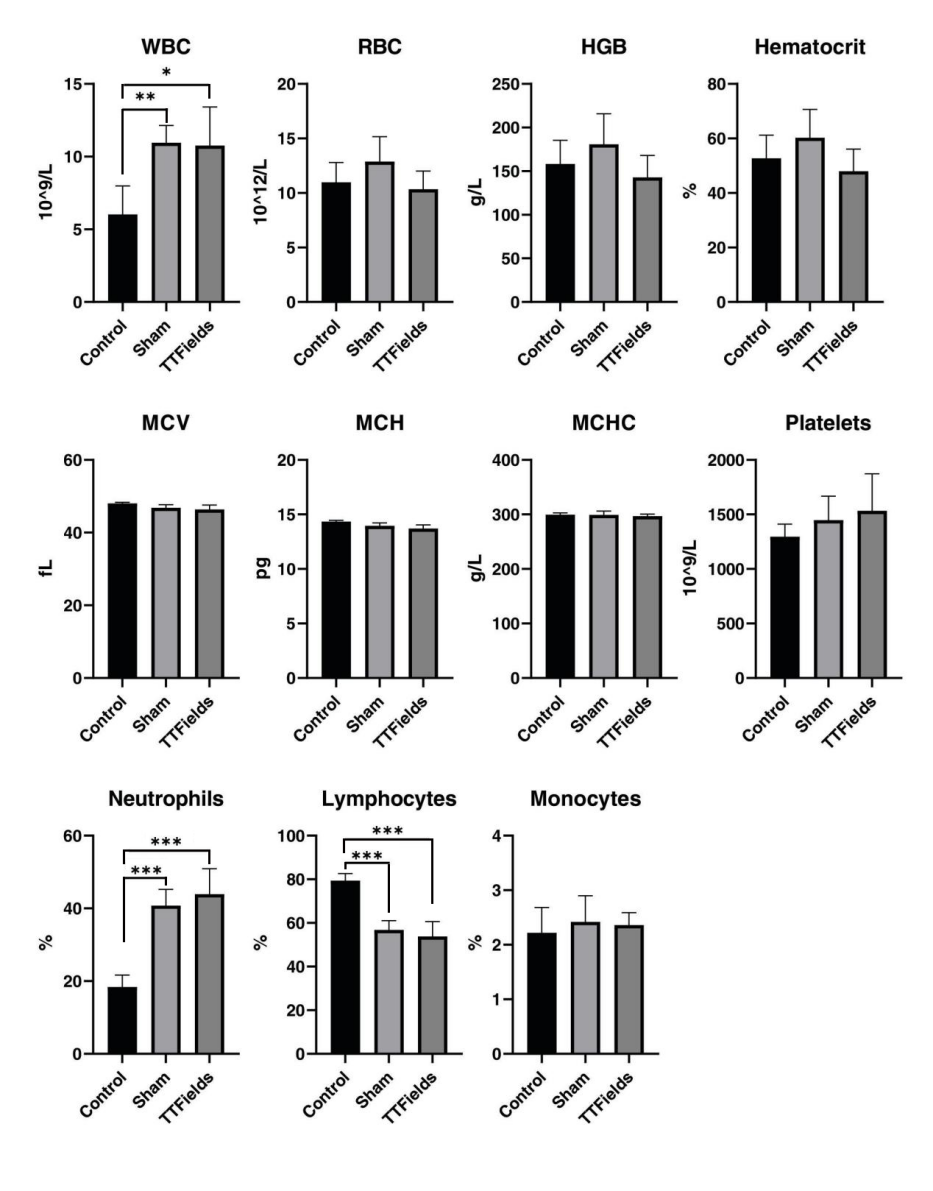
**

**Supplementary Figure 3 The blood routine indexes of the three groups of healthy mice after 14 days of TTFields.** (WBC: white blood cells; RBC: red blood cells; HGB: hemoglobin Hematocrit: hematocrit; MCV: average red blood cell volume; MCH: average red blood cell hemoglobin content; MCHC: average red blood cell hemoglobin concentration platelets; Neutrophils: percentage of neutrophils; Lymphocytes: percentage of lymphocytes; Monocytes: percentage of monocytes).

**Supplementary Figure 4**


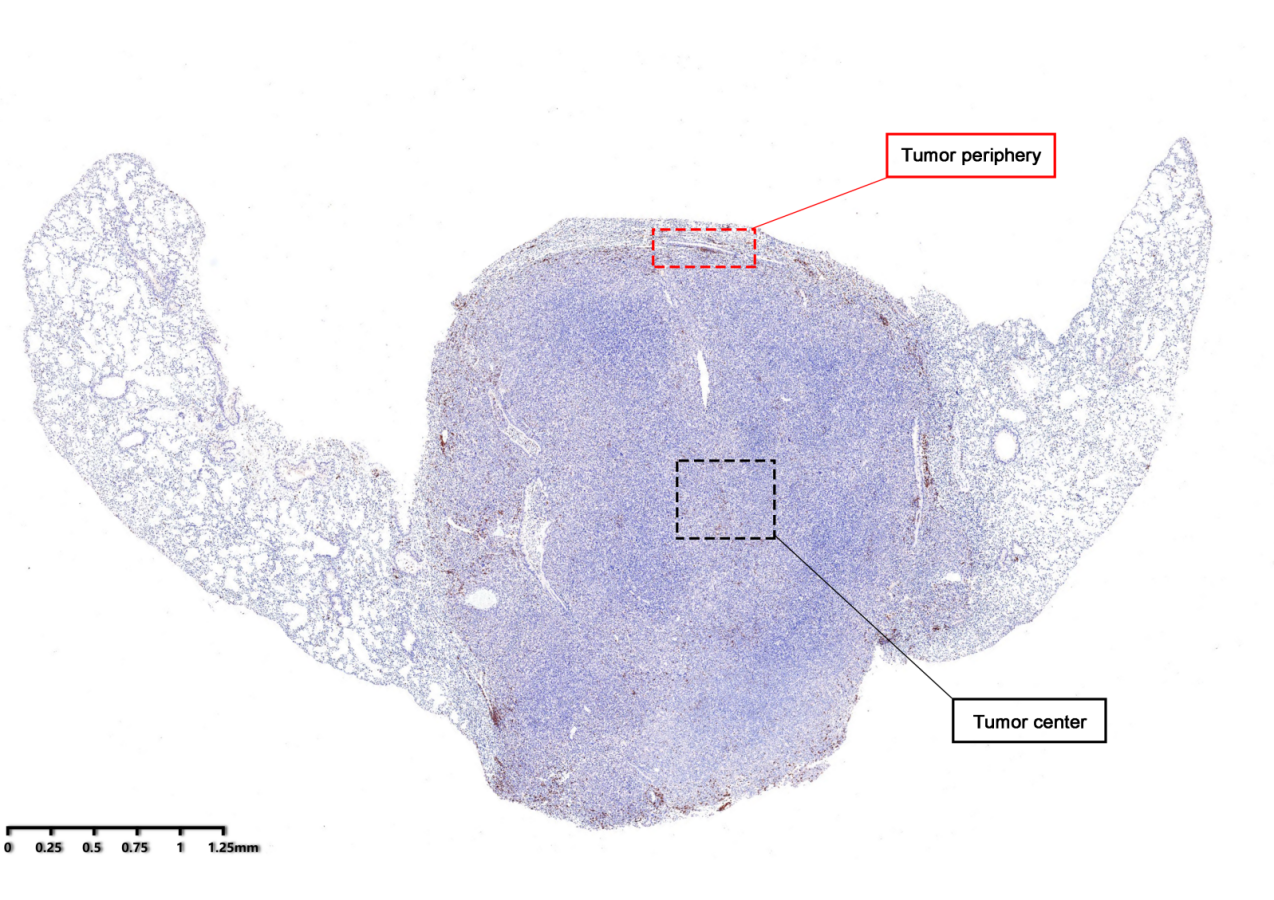


**Supplementary Figure 4 Schematic diagram of the tumor center and periphery.**

**Supplementary Figure 5**

**
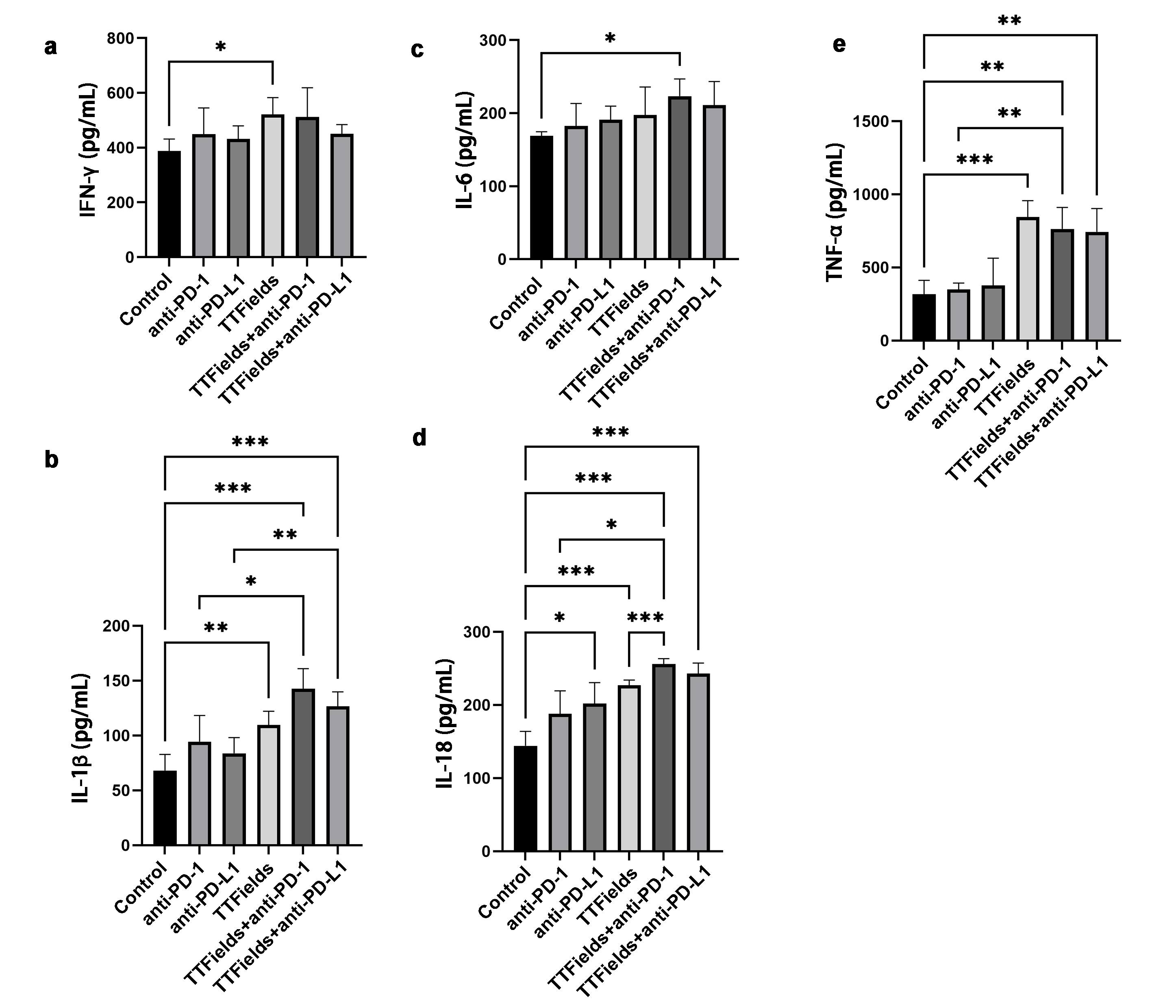
**

**Supplementary Figure 5 TTFields induce cytokine secretion in vivo.**

Cytokine secretion of (a) IFN-γ, (b) IL-1β, (c) IL-6, (d) IL-18 and (e) TNFα in the tumor tissue of every group were detected by ELISA. (n = 6, *p < 0.05, **p < 0.01, ***p < 0.001)

**Supplementary Figure 6**

**
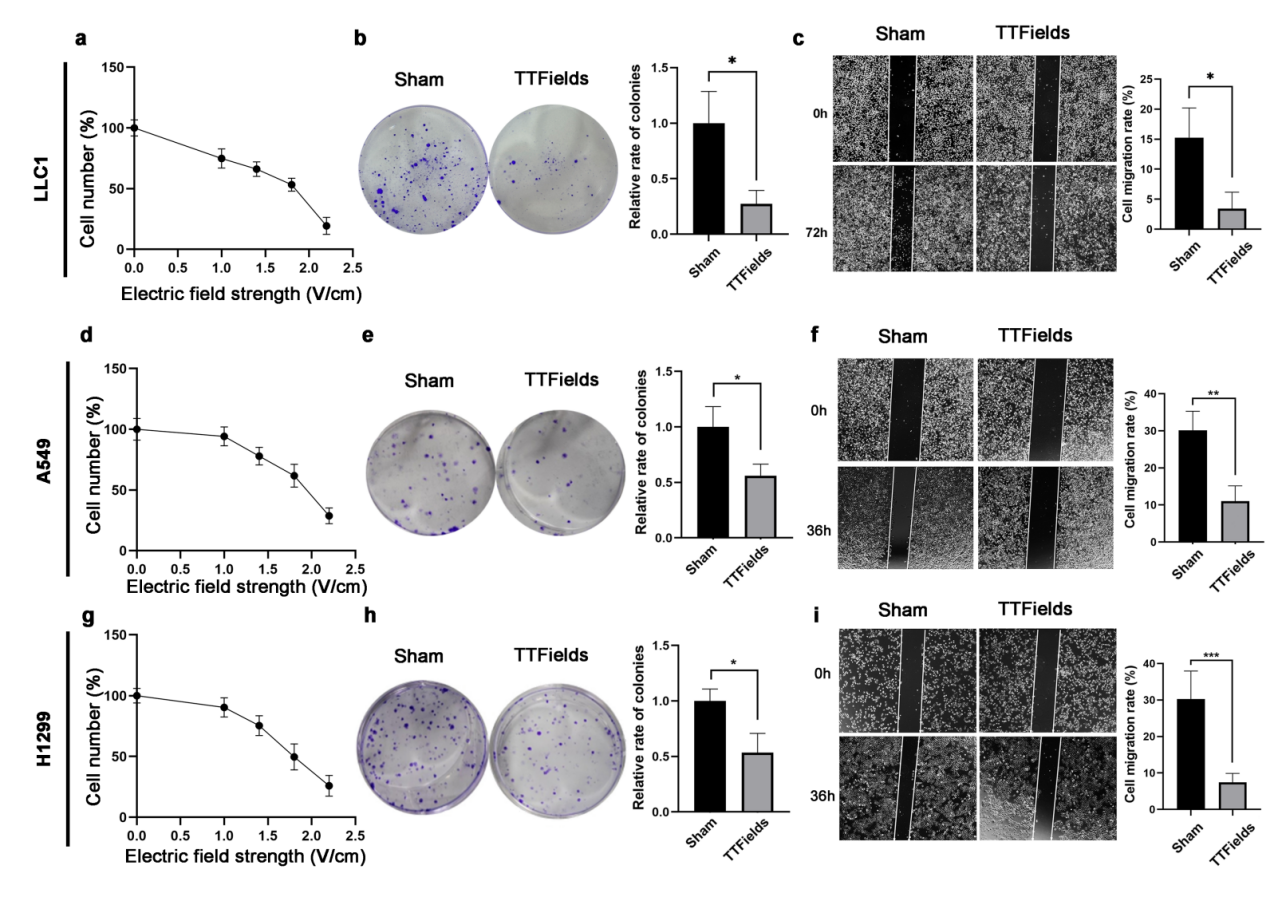
**

**Supplementary Figure 6 TTFields significantly inhibited the viability of NSCLC cells in vitro.**

Intensity-dependent effect, examined for (a) LLC1, (d) A549 and (g) H1299 cells treated with TTFields (1.0~2.2 V/cm) for 72 h at 150 kHz (n = 6). Clonogenicity examined for (b) LLC1 (1.42 ± 0.09 V/cm), (e) A549 (1.50 ± 0.04 V/cm) and (h) H1299 (1.39 ± 0.04 V/cm) cells treated with TTFields for 72 h at 150 kHz (n = 3). The migration ability of (c) LLC1 (1.79 ± 0.14 V/cm, 72 h), (f) A549 (1.70 ± 0.10 V/cm, 36 h) and (i) H1299 (1.73 ± 0.06 V/cm, 36 h) cells was analyzed using the wound healing assay(n = 3). (*p < 0.05, **p < 0.01, ***p < 0.001)

**Supplementary Figure 7**

**
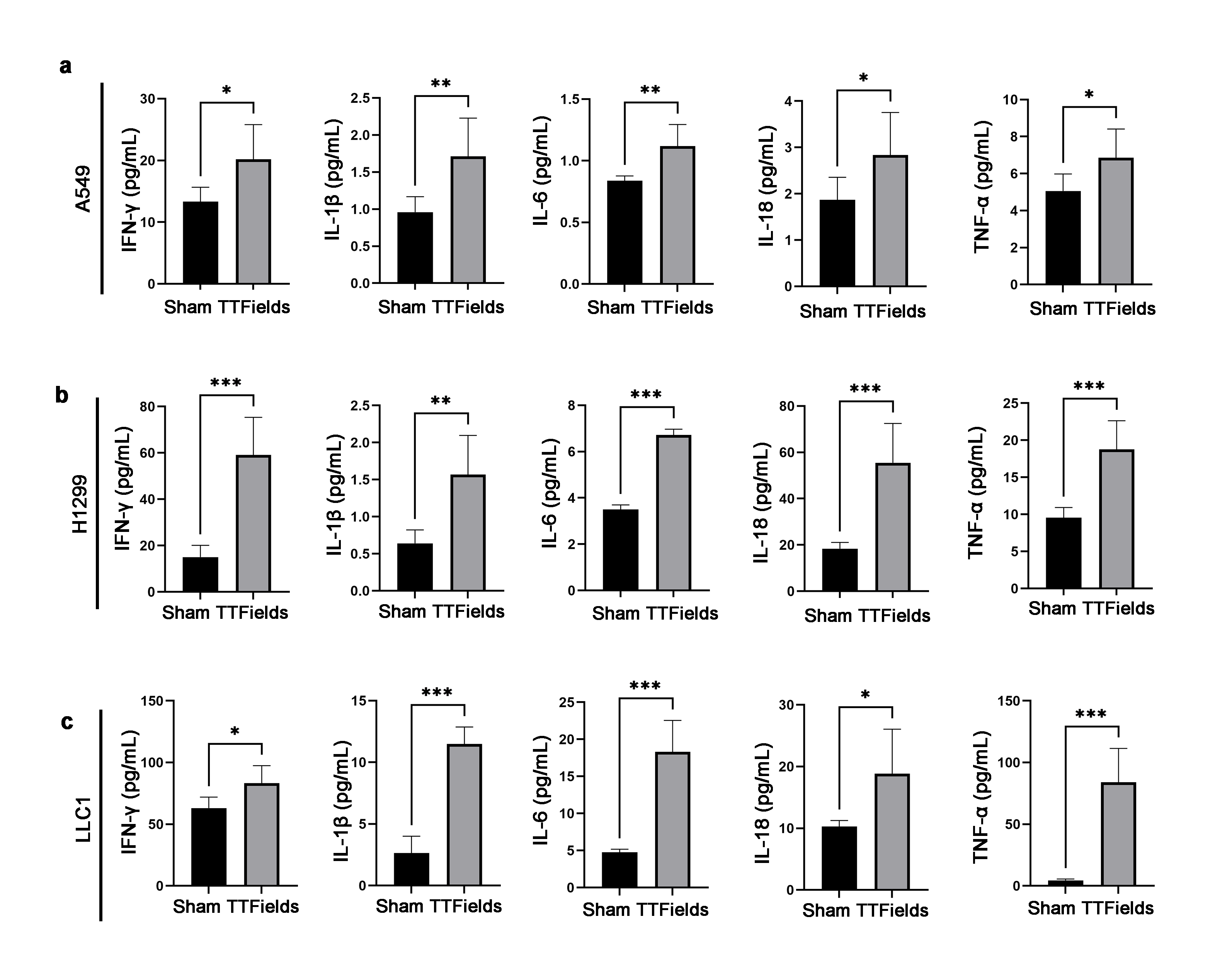
**

**Supplementary Figure 7 TTFields induce cytokine secretion in vitro.**

TTFields induce cytokine secretion in (a) A549, (b) H1299 and (c) LLC1. Secreted IFN-γ, IL-1β, IL-6, IL-18 and TNF-α in the culture supernatant of tumor cells with TTFields treatment or not were detected by ELISA. (n = 6, *p < 0.05, **p < 0.01, ***p < 0.001)

**Supplementary Figure 8**

**
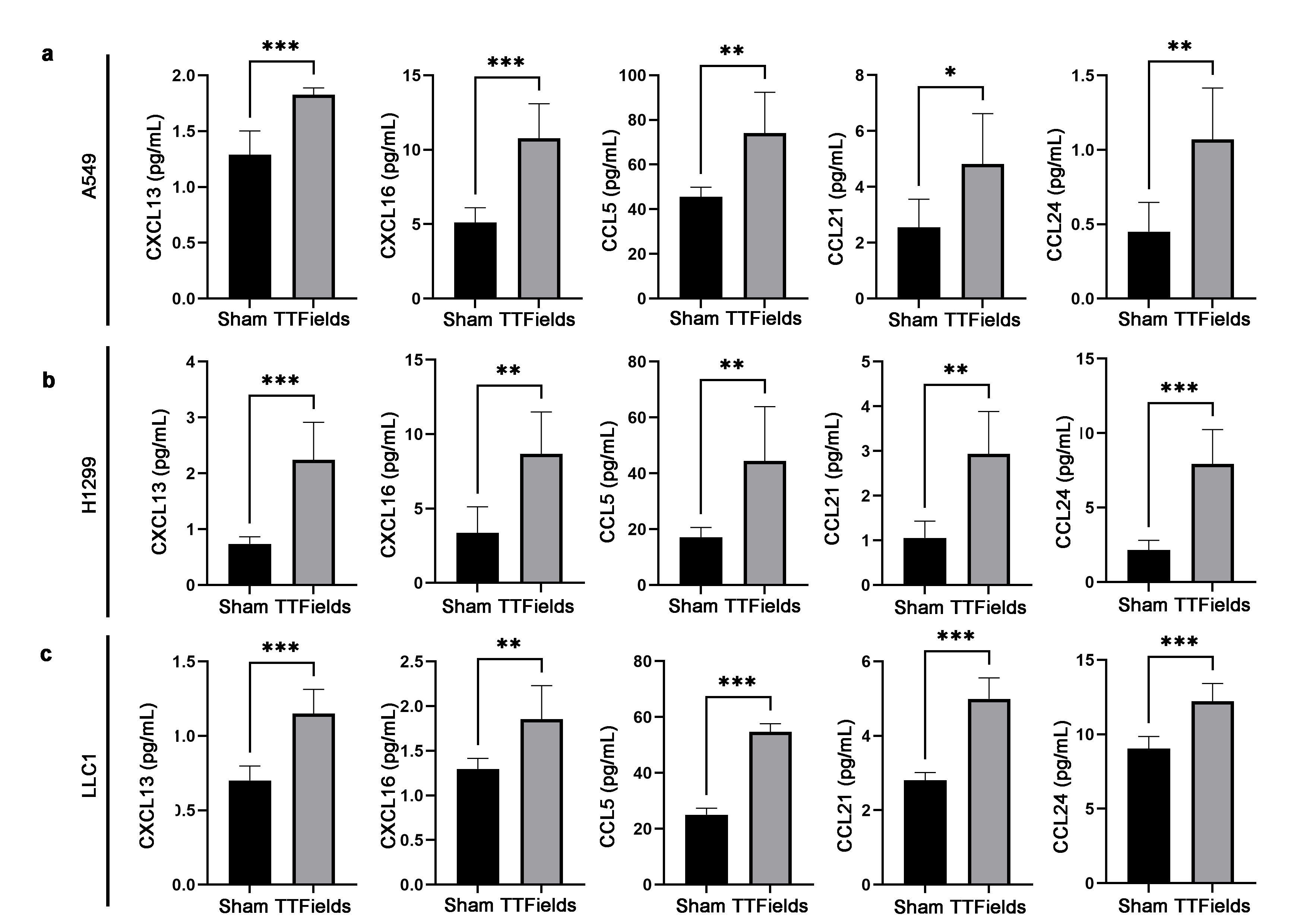
**

**Supplementary Figure 8 Secretion of chemokines in NSCLC cells in vitro.**

TTFields induce chemokine secretion in (a) A549, (b) H1299 and (c) LLC1. Secreted CCL21, CCL24, CXCL13, CCL5 and CXCL16 in the culture supernatant of tumor cells with TTFields treatment or not were detected by ELISA. (n = 6, *p < 0.05, **p < 0.01, ***p < 0.001)

**Supplementary Figure 9**

**
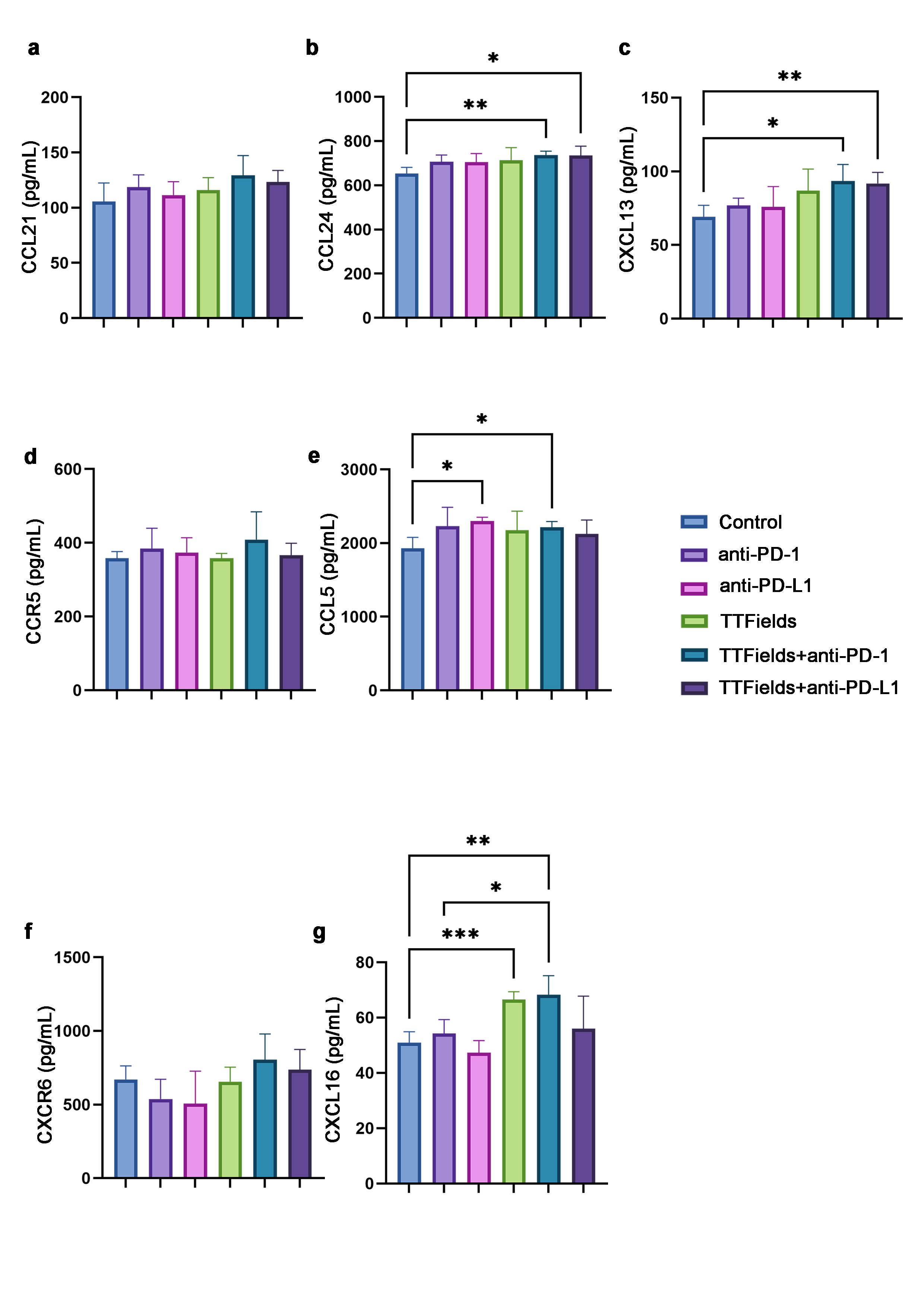
**

**Supplementary Figure 9 Expression of chemokines and their receptors in NSCLC mice tumors tissue.**

Expression levels of chemokines and their receptors, including (a) CCL21, (b) CCL24, (c) CXCL13, (d) CCR5, (e) CCL5, (f) CXCR6 and (g) CXCL16, in tumor tissue of every group were detected by ELISA. (n = 6, *p < 0.05, **p < 0.01, ***p < 0.001)

**Supplementary Figure 10**


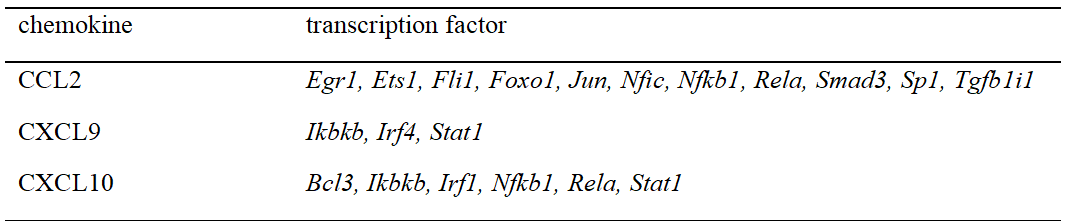


**Supplementary Figure 10 The transcription factors regulate the chemokines CXCL9/10 and CCL2.**

TRRUST database (https://www.grnpedia.org/trrust) was used to predict the transcription factors regulating the chemokines CXCL9/10 and CCL2.

**Supplementary Figure 11**

**
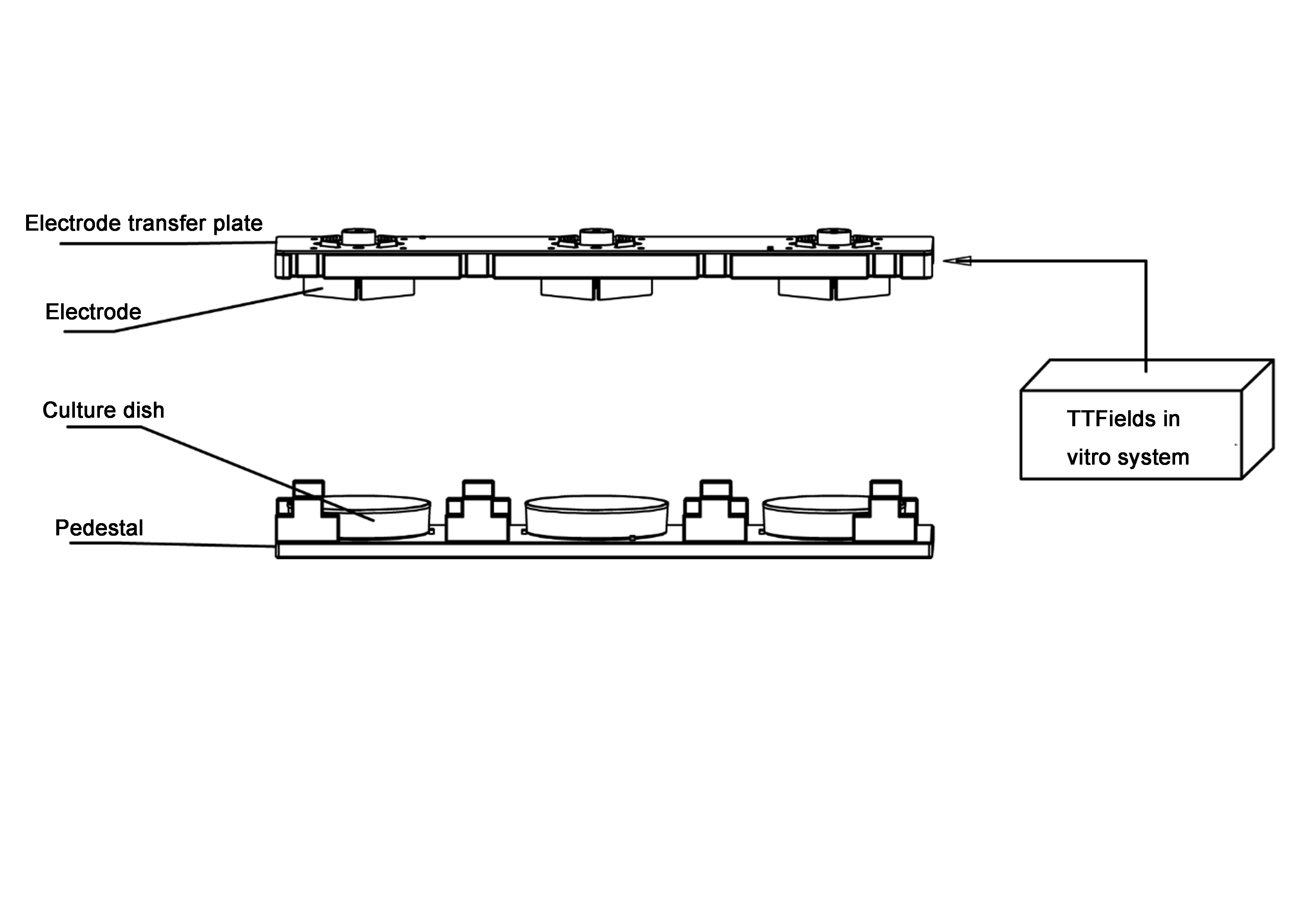
**

**Supplementary Figure 11 The diagram of TTFields equipment.**
